# Mechanical properties of human hepatic tissues to develop liver-mimicking phantoms for medical applications

**DOI:** 10.1007/s10237-023-01785-4

**Published:** 2023-12-10

**Authors:** Aicha S. Lemine, Zubair Ahmad, Noora J. Al-Thani, Anwarul Hasan, Jolly Bhadra

**Affiliations:** 1 Department of Mechanical and Industrial Engineering, College of Engineering, Qatar University, 2713, Doha, Qatar; 2 Qatar University Young Scientists Center (QUYSC), Qatar University, 2713, Doha, Qatar; 3 Center for Advanced Materials (CAM), Qatar University, PO Box 2713, Doha, Qatar

**Keywords:** Liver, Phantoms, Mechanical properties, Tissue-mimicking, Viscosity, Elasticity

## Abstract

Using liver phantoms for mimicking human tissue in clinical training, disease diagnosis, and treatment planning is a common practice. The fabrication material of the liver phantom should exhibit mechanical properties similar to those of the real liver organ in the human body. This tissue-equivalent material is essential for qualitative and quantitative investigation of the liver mechanisms in producing nutrients, excretion of waste metabolites, and tissue deformity at mechanical stimulus. This paper reviews the mechanical properties of human hepatic tissues to develop liver-mimicking phantoms. These properties include viscosity, elasticity, acoustic impedance, sound speed, and attenuation. The advantages and disadvantages of the most common fabrication materials for developing liver tissue-mimicking phantoms are also highlighted. Such phantoms will give a better insight into the real tissue damage during the disease progression and preservation for transplantation. The liver tissue-mimicking phantom will raise the quality assurance of patient diagnostic and treatment precision and offer a definitive clinical trial data collection.

## Introduction

The liver is the largest gland/organ inside the human body, with an average dimension of 8 cm by 16 cm by 28 cm (Ahmad et al. 2020a, b). It is located under the rib cage on the right side of the abdomen within the human body (Ahmad et al. 2020a, b). It acts as the metabolism center for vitamins and nutrient production, as well as excretion of waste metabolites. It also functions as the body’s energy reservoir by storing glycogen (Casciaro et al. 2009). In addition, it is responsible for numerous vital functions such as detoxifying substances, assisting digestion, storing iron, making immune factors, maintaining the hormonal balance, regulating blood clotting, and filtering venous blood (Pellicer-Valero et al. 2020; Umale et al. 2013). However, its functions are affected by a number of pathologies such as hepatitis, liver fibrosis, hepatocellular carcinoma (HCC), liver cirrhosis, and fatty liver disease (Hosseini et al. 2019). These pathologies could result in the total loss of liver functions leading to human death within a matter of minutes to days (McGarry et al. 2020).

**Fig. 1:**
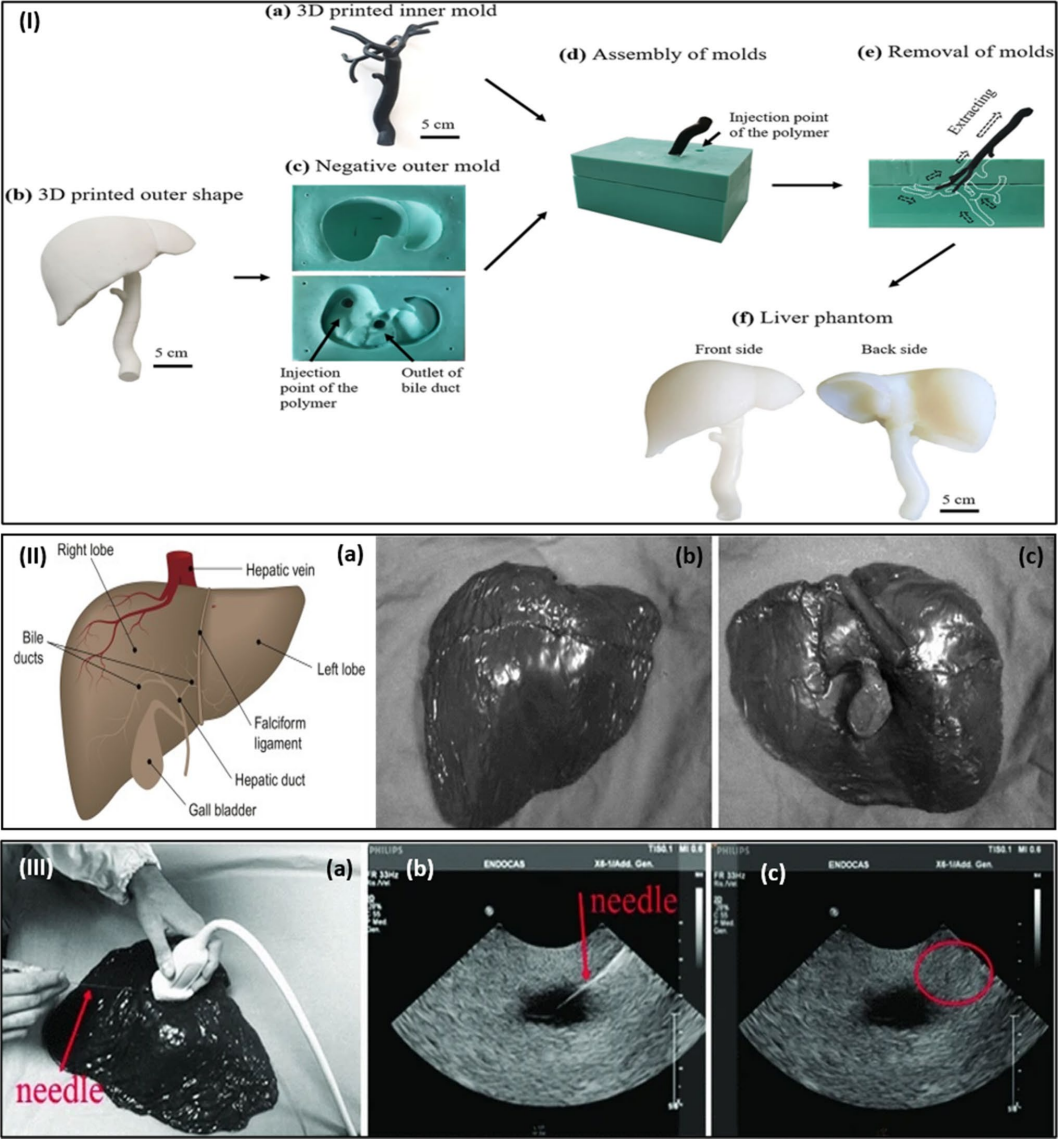
**I** Typical fabrication stages of liver phantom: (a) inner mold printed in 3D by soft material, (b) outer liver shape printed in 3D with a rigid material, (c) negative outer mold consisting of inlet injection point of polymer and outlet of the bile duct, (d) assembly of outer and inner molds through pouring a liquid polymer into the mold, (e) extracted inner mold from outer mold, and (f) liver phantom is molded (Tan et al. 2021), with permission of Springer Nature, copyright 2021. **II** The (a) Liver anatomy and external morphology of liver phantom: (b) Front and (c) back surfaces; **III** The Liver biopsy simulation on liver phantom through the (a) needle’s insertion, (b) visible perfectly on ultrasound (US) scan plane, and (c) reaching the target quickly. No track of the biopsy needle in the parenchyma after its removal due to the self-healing properties of phantom liver material (Pacioni et al. 2015), with permission of Springer Nature, copyright 2015

During the past decade, global attention has increased toward enhancing clinical ethics and adopting simulation stages, particularly in clinical training for interventional and diagnostic trials (Tan et al. 2021), which in return facilitates an adequate training experience for clinical trainees toward improving and promoting medical practices (Bienstock and Heuer 2022). Nowadays, using phantoms as simulators has enhanced the learning experience and clinical ethics (Fu et al. 2013). The phantoms comprise human tissue-mimicking materials (TMMs) fabricated following a typical workflow to be equivalent to actual human tissues (Opik et al. 2012). Figure 1I shows the standard workflow for fabricating liver phantom using 3D printing. A recent study has adopted this workflow for developing a durable liver phantom based on a hybrid simulator consisting of silicone polymer mixed with additives (Tan et al. 2021). The resulting phantom has an external morphology matching that of the human livers, as illustrated in Fig. 1II (Marchesseau et al. 2017). It has been used to simulate biopsies and exhibit an anatomically realistic ultrasound liver phantom mimicking the natural liver anatomy, as shown in Fig. 1III (Pacioni et al. 2015). In addition, the phantom material showed self-healing properties after biopsy needle removal from parenchyma. Such phantom is required for novices’ training on liver ultrasound in interventional and diagnostic procedures (Pacioni et al. 2015).

Realistic mechanical properties can help to advance radiation therapy techniques, for example, using magnetic resonance imaging (MRI) or four-dimensional computed tomography (4D CT) to assess an organ displacement during the response to the respiratory motion (Pi et al. 2021). In addition, the prior knowledge of the liver’s mechanical behavior will enhance the predictive abilities of algorithms for tumor localization (Tian et al. 2015). For instance, liver palpation is a standard screening procedure for identifying hepatic diseases (Leitão et al. 2017). It combines with intra-operative ultrasonography for detecting liver tumors (Leitão et al. 2017). Elastography provides data on stiffness changes among tissues in the liver, which is vital in controlling primary liver tumors (Yin et al. 2011).

Additional studies have adopted dynamic magnetic resonance elastography in detecting liver fibrosis (Ahmad et al. 2020a, b; Basdogan 2012; Saraf et al. 2007). For example, Nava et al. utilized an aspiration device to measure the static mechanical properties of diseased liver invasively and found that the stiffness of fibrotic tissue is three times that of normal tissues (Nava et al. 2004). In addition, Maccabi et al. used an impact hammer to examine the dynamic properties associated with liver tissues affected by liver fibrosis (Maccabi et al. 2018). They detected a rise in human liver elastic storage modulus (E′) when subjected to growth in fibrosis level, which is recognizable via histological scoring. In addition, the influence of collagen alignment on both structural and mechanical behaviors of liver tissues in response to compression was examined (Maccabi et al. 2018). The study concluded a significant variance between collagen alignments and stress relaxation responses.

Herein, this paper will review the qualitative and quantitative methods used in examining the correlation between the liver tissues’ histological and mechanical properties toward understanding the mechanisms of tissue-damaging throughout the evolution of liver diseases. In this regard, the present paper addresses the following:

- Standard protocols and tailored guidelines for sample preparation, mechanical testing, and data analysis of liver tissues.
- Monitoring, detecting, and measuring tools for the liver’s mechanical properties.
- Most commonly used mechanical properties to design liver tissue-mimicking phantoms.
- Impact of liver diseases on its mechanical properties.
- Constitutive models for human hepatic tissues.
- Most common materials used to fabricate liver phantoms and their advantages and disadvantages.
- Factors affecting the measurements of liver mechanical properties.

## Mechanical testing of liver tissues to develop mimicking phantoms

The mechanical properties of biological tissues are affected by sample conditions and testing protocols (Kassner et al. 2009). The target liver tissue and engineered tissue mimetic materials characterization must follow the same testing and analysis methods for maintaining testing variables. In vivo or imaging-based methods are preferable to direct ex vivo tests. Still, their drawback in being difficult and expensive, as well technology, is not available to measure some properties in vivo. In ex vivo trials, the isolated tissue blocks undergo direct measurements of mechanical properties in tension, compression, or shear (Kassner et al. 2009). The mechanical testing of the liver is difficult due to the need to maintain specific homogeneity.

**Fig. 2:**
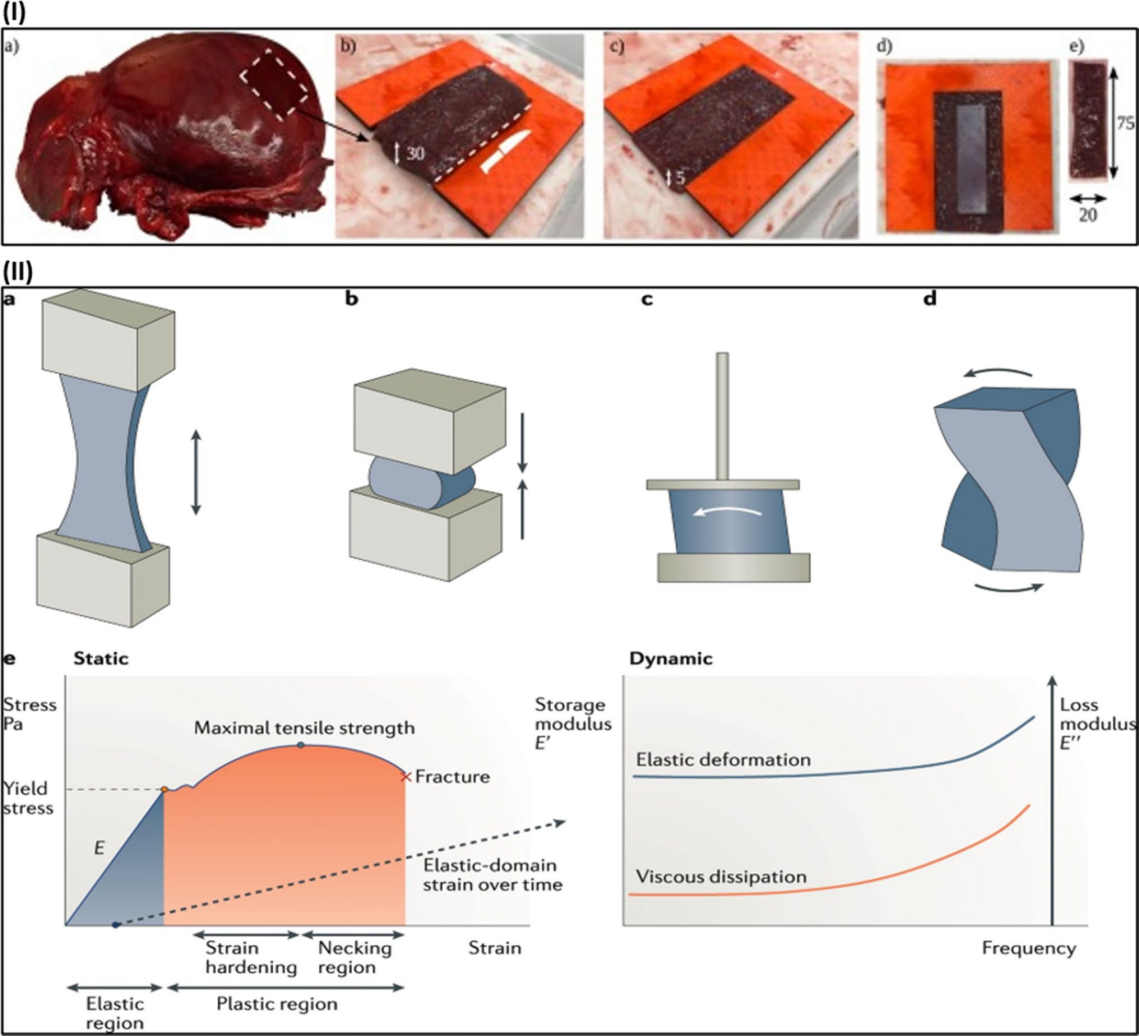
**I** Typical sample preparation through cutting out the (a) whole human liver organ into (b) block of hepatic tissue for placing onto the 3D-printed cutting guide to extract (c) thin liver tissue layer from the block. Then (d) placing a rectangular stencil onto the tissue layer to (e) cutting a sample from the tissue layer in dimensions of 75 × 20 × 5 mm (Estermann et al. 2021), with permission of Elsevier Ltd., copyright 2021. **II** Standard mechanical analysis deformations include (a) tensile, (b) compressive, (c) shear, and (d) torsion. (e) The typical static and dynamic deformations (Guimarães et al. 2020), with permission of Springer Nature, copyright 2020

Figure 2I shows the typical preparation stages for a rectangular tensile testing specimen based on a human liver sample (Estermann et al. 2021). The Glisson capsule is removed before the sample preparation to get consistent specimens with a high proportion of parenchyma tissues (Karimi and Shojaei 2018). In addition, avoiding the bile ducts and large blood vessels ensures relatively homogenous samples. The standard biomechanical testing is either static or dynamic. For static testing, the applied deformations can be tensile, compressive, shear, or torsion (Fig. 2II, (Guimarães et al. 2020)). The slope in the elastic (linear) area of the stress–strain curve corresponds to Young’s modulus (E) (Lemine et al. 2022). The plastic domain of the curve occurs at higher levels of strain, where the material undergoes irreversible and permanent deformations until fracturing.

In dynamic analysis, the dynamic loading test is a prevalent method for identifying the viscoelastic properties of soft tissues dependent on frequency (MacManus et al. 2019; Saraf et al. 2007). This test applies minor episodic strains at variable frequencies onto the tissue sample to record the stress response and produce the loss (E″) and storage (E′) moduli, as shown in Fig. 2 (II, e) (Guimarães et al. 2020). The loss modulus reflects the dissipated energy through the internal structural rearrangements, and the storage modulus is associated with the material's ability to store energy through material elastic deformation (Mulabecirovic et al. 2018a). Relaxation studies have also been used in biomechanics literature to test the soft tissues’ viscoelastic properties depending on time (Morr et al. 2021a; Wang and Shi 2020). In relaxation studies, a strain is applied and held constant, resulting in the stress decaying exponentially until the steady-state value is attained. It is also recognized with a time-dependent relaxation modulus (Mattei and Ahluwalia 2016).

Significant efforts have introduced standardized methods for examining tissue-engineered products (Sorrentino et al. 2020). The American Society for Testing and Materials (ASTM) has developed ASTM testing standards to enhance these products’ consistency, safety, and quality (Johnson et al. 2021; Sorrentino et al. 2020). These standards are explicitly not designed to conduct mechanical tests on biological tissues like human tissues, but they could still guide appropriate testing (McGarry et al. 2020). Specific standards for acoustic properties characterization of liver clinical trials and some mechanical properties as Young’s modulus are provided by the American Institute of Ultrasound in Medicine (AIUM) (Greenbaum et al. 2007). Table 1 summarizes living tissues’ standard mechanical characterization techniques to investigate different mechanical properties based on the proper ASTM protocols for ex vivo or in vivo samples. Section 3 will discuss the effect of these properties in the diagnosis and treatment of hepatic tissues.

**Table 1:** Common mechanical characterization techniques of living tissues to estimate Young’s modulus (E), viscoelastic gain (E′) and loss (E′′) moduli, shear loss (G′′), and storage (G′) moduli based on the ASTM standard testing methods

Technique	Working principle	Modulus	Sample	ASTM	Ref
Tensile deformation	A standard stress–strain analysis uses uniaxial stress to stretch the sample and establish a relationship with the yield strain	E (elastic)	Ex vivo tissue	D3039M, D3039, D1708, D1623, D882, D638, D412	Karimi and Shojaei (2018)
Compressive deformation	It is a standard stress–strain analysis through the application of uniaxial stress to compress the sample and establish a relationship with the yield strain. The size of the compressor is similar to or larger than the sample	E (elastic)	Ex vivo tissue	D1621, D695	Karimi and Shojaei (2018), Maccabi et al. (2018)
Shear rheometry	The resulting strain quantifies through the application of low-amplitude oscillatory shear stress	G′′, G′ (viscoelastic, shear)	Ex vivo tissue	D5279	Mulabecirovic et al. (2018b), Zhu et al. (2016)
Dynamic mechanical analysis (DMA)	The deformation of the sample is through cycles of tensile and compressive	E′′, E′(viscoelastic)	Ex vivo tissue	D5026, D5024	Zhang et al. (2017)
Indentation	The tissue sample is indented with a smaller probe than the sample and has a defined geometry to calculate the relationship between the probe load and indentation depth	E (elastic)	Ex vivo tissue	E2546-15	Maccabi et al. (2018)
Atomic force microscopy (AFM)	The AFM relies on shear rheology or nanoindentation	G′, G′′ (shear), E (indentation)	Dry/wet ex vivo tissue	–	Tian et al. (2015)
Micropipette aspiration	The technique forms a relationship between the sample aspiration volume and the aspiration pressure	E (elastic)	Ex vivo tissue	–	Mazza et al. (2008)
Ultrasonic shear wave elastography (USWE)	Shear waves generate across the tissue through ultrasonic pulses, and the velocity of the waves is measured to derive Young’s modulus of tissues	E (elastic)	In vivo tissue	–	Imajo et al. (2021), Yeh et al. (2002)
Magnetic resonance elastography (MRE)	Visualizing tissue deformation through magnetic resonance occurs from the shear waves introduced into the tissue generated from external vibrations	G′′, G′ (viscoelastic, shear)	In vivo tissue	–	Cournane et al. (2012), Garczyńska et al. (2020)

## Mechanical properties of liver tissues to develop mimicking phantoms in hepatic diseases diagnosis and treatment

### Elasticity

Elasticity indicates the material’s ability to recover its original shape and size when subjected to stress or deformation (Kim et al. 2013). The mechano-sensitivity of parenchymal and non-parenchymal cells shows altering their behavior with changes in liver stiffness (Hoodeshenas et al. 2019). Mechanical stiffness also contributes to driving the myofibroblastic differentiation of hepatic stellate cells (HSCs) (Yin et al. 2011) and portal fibroblast (Cafarelli et al. 2017). In the cellular micro-environment, the principal biomechanical cue is the extracellular matrix (ECM) stiffness (Evans et al. 2013). It regulates cell behavior and changes during tissue fibrosis in response to different pathologies like non-alcoholic fatty liver disease and fibrosis (Boursier et al. 2016; Ijima et al. 2018). These pathologies affect the stiffness of the hepatic tissues at cellular levels, regional, or whole organs (Boursier et al. 2016).

**Fig. 3:**
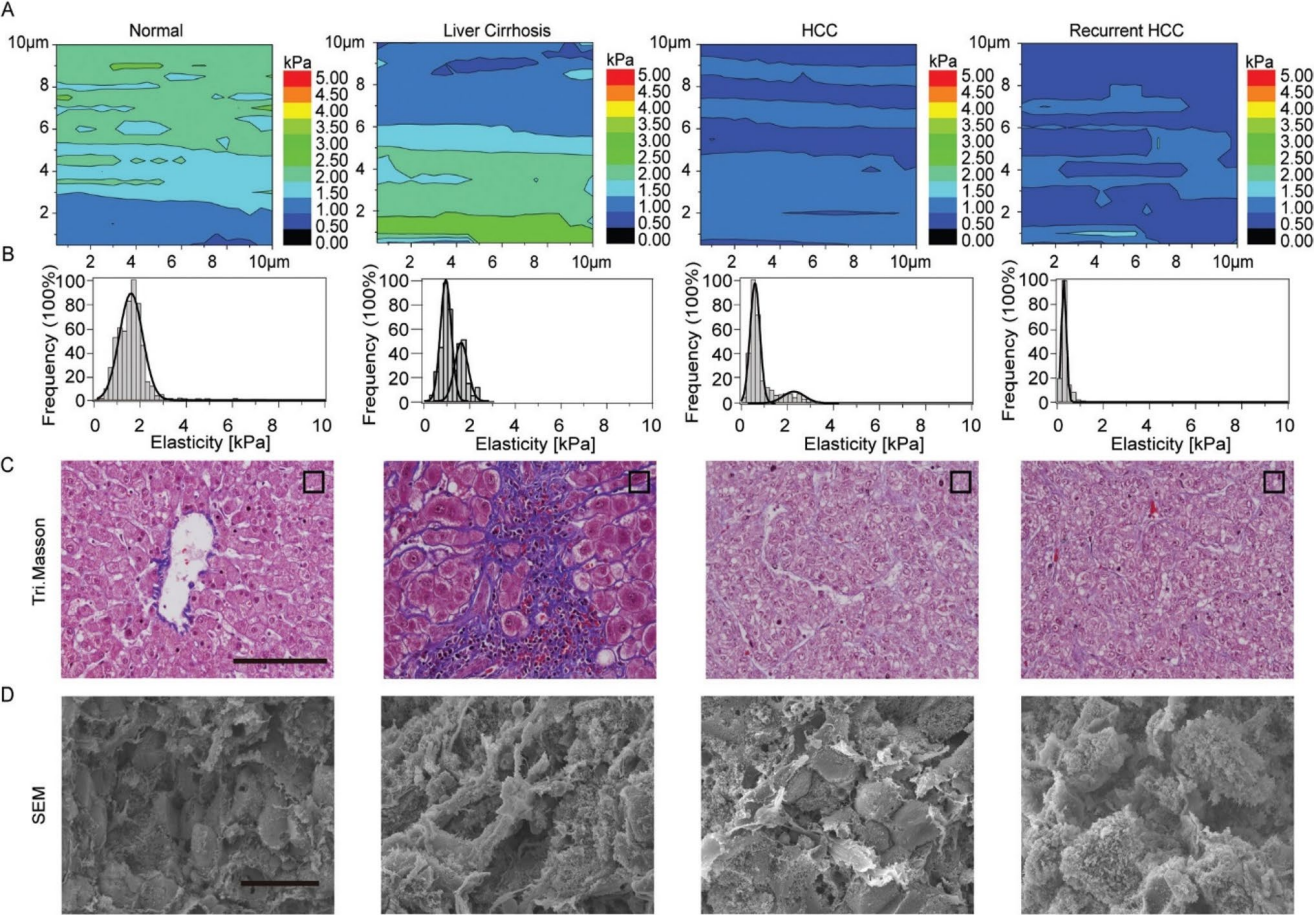
Nanomechanical properties changes during liver cancer progression. **A** Elasticity maps in 10μmx10μmx100pixels, **B** elasticity distributions, **C** Tri. Masson staining images and **D** SEM images of liver cancer tissues at various stages from (left) normal, liver cirrhosis, HCC, to (right) recurrent HCC, respectively. The SEM and Tri. Masson images are in scale bars of 50 μm (Tian et al. 2015), with permission of the Royal Society of Chemistry, copyright 2015

Liver cirrhosis is a diffuse nodular regeneration enclosed by dense fibrotic septa besides successive extinctions of parenchyma and collapses of liver structures (Mueller 2010). It results from fibrogenesis and necroinflammation (Mueller 2010). It might develop to hepatocellular carcinoma (HCC) cancer, which is the most severe adult death as it is responsible for the death of over 12,000 adults within a year in the United States (US) (Makhamrah et al. 2019). Figure 3 shows the changes in the liver’s nanomechanical properties throughout cancer progression at typical liver cirrhosis, HCC, to recurrent HCC (Tian et al. 2015). 1–3% of diagnosed patients with liver cirrhosis have developed HCC annually (Tian et al. 2015). The liver tissues could alter their nanomechanical properties during different stages of HCC. The bimodal elasticity distribution indicates that there is a health and a diseased group. In healthy liver tissues, the elasticity distribution is characterized with the lowest elasticity peak (LEP) ranging from 0.91kPa to 1.55kPa, as demonstrated in Figs. 3A, B (Tian et al. 2015). The LEP is frequently reported as the mechanical fingerprint to evaluate the malignancy in living cells (Tian et al. 2015).

The hematoxylin stains purplish-blue the cell nuclei, while the eosin stains pink the cytoplasm and ECM (Hoodeshenas et al. 2019). The Masson’s trichrome (Tri. Masson) is used to distinguish between collagen and smooth muscle in tumors, as well as the increase of collagen in diseases like cirrhosis (Cabibi et al. 2015). In Fig. 3 (C, regular column), Tri. Masson and HE staining illustrate that the liver tissues have organized well with less fibroconnective tissue and packed hepatocytes. The heterogeneous distribution in elasticity values can show up to five peaks centering in the range of 4.65–11.50 kPa. It could be attributed to the nanomechanical properties associated with the blood vessels in the portal area (Tian et al. 2015). These values are comparable to benign fibroadenomas of 3.68 ± 1.92 kPa (Yin et al. 2011).

In liver cirrhosis tissues, two or more elasticity peaks might be caused by intrahepatic scaffold turbulence (Fig. 3B). However, there are no significant differences in LEPs compared to normal liver tissues. The higher elasticity peak (HEP) increased, and the elasticity distribution broadened as the local fibrogenesis became severe, as shown in Fig. 3 (A and B, liver cirrhosis column). Many tissues have a stiffness reaching a maximum of 16 kPa, indicating the occurrence of abundant ECM within the paraneoplastic tissue (Ijima et al. 2018). The staining in Tri. Masson images, as shown in Fig. 3C, illustrates the proliferation of ECM distributed around the hepatocytes across the tissue. The subsequent SEM analysis has confirmed these cues, as presented at the bottom of the liver cirrhosis column in Fig. 3D. Furthermore, it reported that liver cancer’s driving force is chronic hepatic fibrosis (Yin et al. 2011). Consequently, the mechanical microenvironment undergoing disordered homeostasis could lead to malignant transformation, raising the need to investigate further the correlation between carcinogenesis and ECM’s mechanical properties (Yin et al. 2011).

The HCC cancer tissue showed an elasticity distribution in the form of two distinct peaks centering at 0.6 kPa and 2.1 kPa, as shown in Fig. 3 (A and B, HCC column). A decrease in LEP characterizes the progression to a malignant state compared to normal and cirrhotic liver tissues. The SEM and Tri. Masson staining, as shown in Fig. 3 (C and D, HCC column), have revealed the origin of such mechanical features. The HCC becomes softer compared to other malignant epithelial neoplasms due to decreased tumor stroma and cell variations in mechanical phenotype (Mueller 2010). Nanomechanics of recurrent cancer has displayed a unimodal peak centered at 0.45kPa within its elasticity histogram, similar to LEP of HCC, as shown in Fig. 3 (A and B, recurrent HCC column). In addition, the SEM images in Fig. 3 (D, recurrent HCC column) illustrate large numbers of plentiful microvillus-like protrusions on the surface of recurrent cancer cells requiring curative resection (Kassner et al. 2009).

**Fig. 4:**
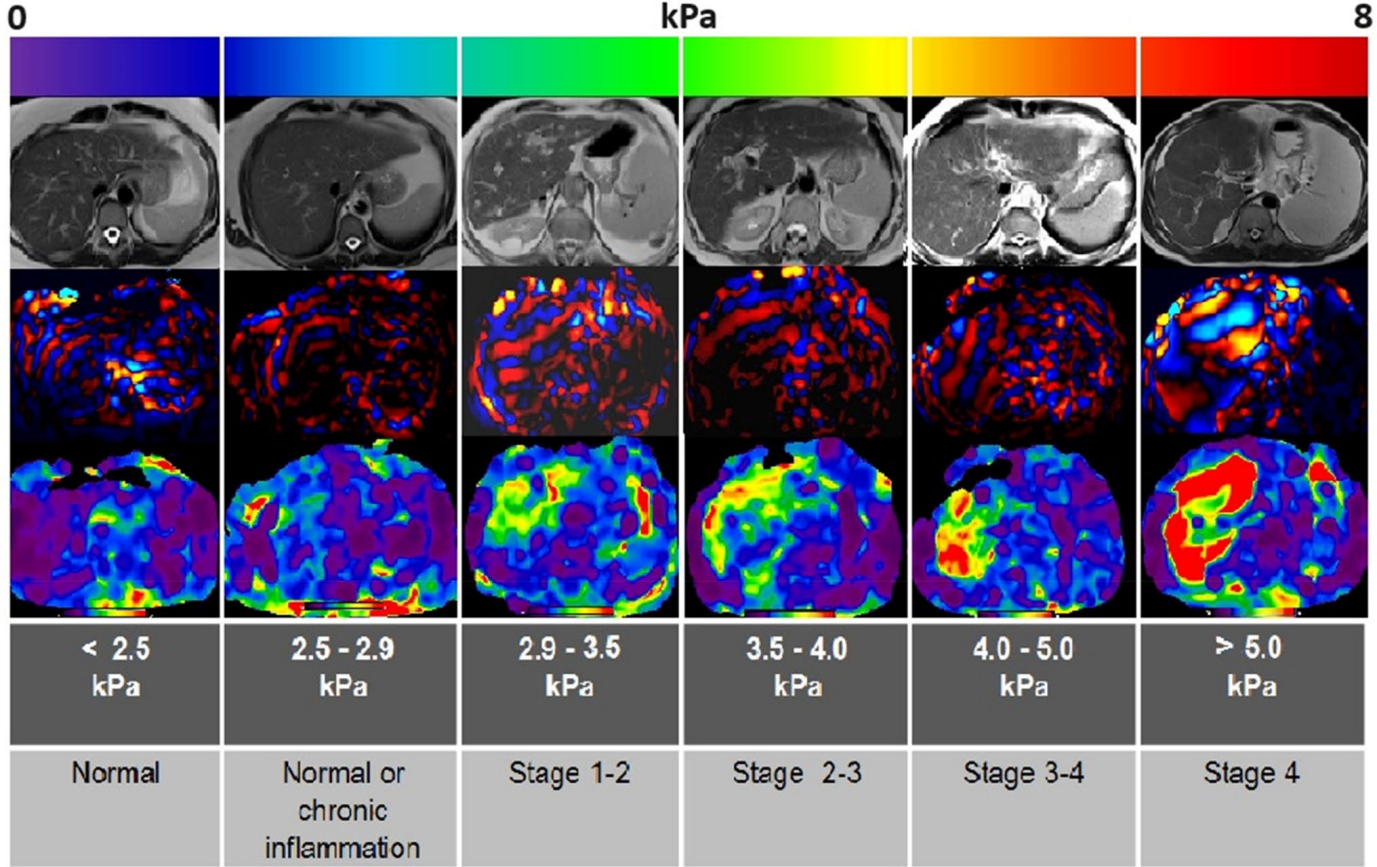
Effect of different hepatic fibrosis stages on the MRE-measured liver stiffness. The relative tissue stiffness is shown in the color-coded elastography on a color scale from 0 kPa (softest with purple color) to 8 kPa (hardest with red color) (Akkaya et al. 2018), with permission of PMC Publications, copyright 2018

The elastography imaging technique also inspects the mechanical properties of natural liver tissues (Pasyar et al. 2020). The magnetic resonance elastography (MRE) imaging technique utilizes the changes of shear wave wavelengths passing through the liver in measuring the tissue viscoelasticity (Akkaya et al. 2018). The average liver stiffness values from MRE measurements in a healthy human body range from 2.05 kPa to 2.12 kPa, with minor variations for age or gender (Zhang et al. 2017). Thus, the stiffness values of normal livers are commonly lower than 2.5 kPa, as shown in Fig. 4 (Akkaya et al. 2018). The stiffness values more than 2.5 kPa are utilized in MRE measurements to diagnose hepatic fibrosis with high specificity and sensitivity (Akkaya et al. 2018). The stiffness values undergo proportional incrementations with different histologic fibrosis grades differentiated in MRE elastography, as shown in Fig. 4. The early stages of hepatic fibrosis are not detectable using routine imaging techniques, while the liver fibrosis grades are all detected in high sensitivity, exceeding 95% using MRE measurements (Leitão et al. 2017).

## Viscoelasticity

The liver is highly viscoelastic, which can provide a means for clinical diagnostics (Yeh et al. 2015). Liver viscoelasticity depends on the fibrosis stage and other factors like inflammation, congestion, extrahepatic cholestasis, and edema (Sugiura et al. 2019). Different clinical protocols are available to evaluate fibrosis and cirrhosis extent (Crescenzi et al. 2019). The Scheuer classification and METAVIR scale categorize fibrosis into five stages (Cournane et al. 2010). Stage 0 (F0) indicates no fibrosis, stage 1 (F1) is minor fibrosis, stage 2 (F2) is the extension of fibrosis into areas close to the portal vein, stage 3 (F3) is a further extension of fibrosis outside the areas of the portal vein, and stage 4 (F4) is evolving of fibrosis into cirrhosis (Cournane et al. 2010). The F4 is the advanced pathological stage leading to the distortion of hepatic architecture and vasculature (Mueller 2010). Table 2 shows the liver viscosity at stage F4 might reach 3.7Pa.s comparable to 6.7Pa.s at the healthy state (Deffieux et al. 2015; Garczyńska et al. 2020; Zhu et al. 2016).

**Table 2:** Viscoelastic biomarkers of human liver tissues in a healthy state and different fibrosis states using shear wave spectroscopy (SWS) and magnetic resonance elastography (MRE)

Hepatic tissue state	Viscosity (Pa.s)	Testing method	Ref.
Healthy	7.3 ± 2.3	MRE	Idilman et al. (2020)
Healthy	6.7 ± 1.3		Deffieux et al. (2015)
Fibrosis (F0)	2.0 ± 0.8	SWS	Lin et al. (2017)
Fibrosis (F1)	2.3 ± 0.7		Lin et al. (2017)
Fibrosis (F2)	2.6 ± 0.5		Pasyar et al. (2020)
Fibrosis (F3)	2.7 ± 1.9		Mazza et al. (2007)
Fibrosis (F4)	3.7 ± 2.5		Seyedpour et al. (2021)

**Fig. 5:**
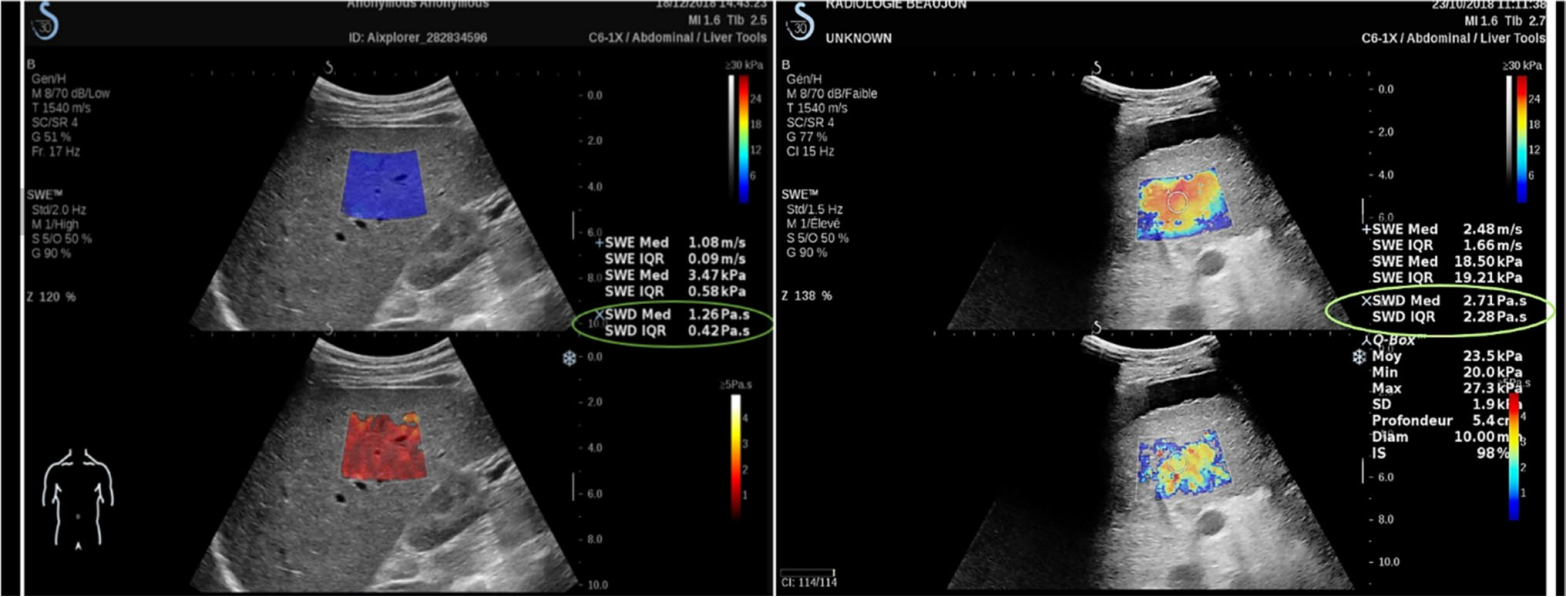
Real-time viscosity measurements via supersonic shear imaging (SSI) AIXPLORER MACH30® on a healthy volunteer (left) and patient volunteer (right) with cirrhotic liver (Rus et al. 2020), with permission of MDPI Publications, copyright 2020

Furthermore, liver viscoelasticity plays a crucial role when elastography contrast is insufficient (Gidener et al. 2021). A proposed approach utilizes liver viscoelasticity to separate the severely rejected transplanted livers from the non-rejected ones (Ijima et al. 2018). Zhang et al. characterized the viscoelastic parameters of the liver without using rheological models but computed through the attenuation of shear wave elastography (AMUSE) (Zhang et al. 2017). The attenuation and shear wave velocity for fifteen transplanted livers have diagnosed patients with severe rejection and revealed a high agreement with biopsy results (Deffieux et al. 2015). Recently, the viscosity biomarker has facilitated supersonic shear imaging (SSI) to release its latest and advanced characterizing device with novel liver features like the viscosity imaging feature (Glińska-Suchocka et al. 2017). Furthermore, utilizing SSI provided more accuracy than from shear wave imaging using transient elastography (TE) from FibroScan (Glińska-Suchocka et al. 2017). Figure 5 displays the imaging of healthy and cirrhotic liver with real-time viscosity values (Rus et al. 2020).

The lack of practical guidance and agreement among the elastography specialist or clinical community on the most suitable rheological model to characterize the soft tissue has put an additional focus on dispersion slope measurements (Idilman et al. 2020). The dispersion slope is a viscosity-related parameter measured through shear wave speed (SWS) imaging to delimit the degree of fibrosis and diagnose necroinflammation and steatosis in non-alcoholic fatty liver disease (NAFLD) (Imajo et al. 2021). NAFLD severe cases might lead to cirrhosis and a vital liver transplant requirement (Hosseini et al. 2019). A set of preliminary studies based on ex vivo and in vivo liver samples from goose, porcine, mouse, and duck have utilized viscoelasticity as a potential biomarker in characterizing fatty liver (Wang and Shi 2020).

Many current publications have emphasized MRE as a technique for detecting and staging liver fibrosis (Gidener et al. 2021). A study has inspected the enhanced liver fibrosis (ELF) index’s performance features compared to MRE and concluded that the ELF index had shown specific markers and high sensitivity to cirrhosis compared to MRE (Suh et al. 2014). A posterior study on a cohort of 102 patients undergone both liver biopsy and MRE (Crescenzi et al. 2019). The study aims to assess the association between fibrosis progression in NAFLD and increased liver stiffness on MRE. It is concluded that the 15% of liver stiffness increase on MRE probably correlated to the histological fibrosis progression. Therefore, viscosity imaging is a noninvasive and essential biomarker for further information on liver pathology (Crescenzi et al. 2019). However, a precise viscosity quantification is still a challenging inquiry, and the viscoelastic model selection determines the accuracy of the outcomes.

## Acoustic impedance and attenuation

The acoustic impedance is the resistance to the ultrasound waves’ propagation through the tissues (Zell et al. 2007). It results from the speed of sound across the tissue and its density, which varies from one tissue type to another, giving the unique fingerprint of acoustic impedance (Zell et al. 2007). In medical ultrasound, the acoustic impedance becomes evident at the interfaces between dissimilar tissue types (Cournane et al. 2010). Transferring ultrasound waves from one tissue type to another varies depending on the variation in impedance of the two tissues (Cournane et al. 2010). The significant variations in impedance will cause reflection of sound. For instance, the passing of an ultrasound beam through the muscle tissue coming across the bone causes it to reflect off of it due to the differences in tissue density. The impedance increases with tissue densities and shows less sensitivity to increased speed of sound (Cafarelli et al. 2017). Therefore, the high acoustic impedance is better as it will produce superior sound quality for professional medical diagnostics.

In the literature, the acoustic impedance of liver tissues in the human body is around 1.65*10^6^Rayls (Deffieux et al. 2015). It is similar to blood and kidney, lower than bone with 7.8*10^6^Rayls, and higher than lung and fat with 0.18*10^6^Rayls and 1.34*10^6^Rayls, respectively (Nava et al. 2004). Recently reported that liver phantom-mimicking materials based on silicone exhibit a close acoustic impedance to the real human liver, as displayed in Table 3 (Afiqah Bakri et al. 2019; Cafarelli et al. 2017). This table also shows other acoustic properties of the liver and its phantoms as sound speed, density, and attenuation. The density of liver organs and phantoms is 1060–1080 kg/m^3^, while the sound speed is 1540–1000 m/s, respectively. The attenuation of liver phantoms varies widely from the real human liver. The hepatic attenuation is influenced mainly by the accumulation of intracellular vacuoles of triglycerides as hepatic steatosis (Casciaro et al. 2009). Thus, the increased attenuation values are associated with cardiac and alcoholic cirrhosis, as well as the infusion of the hepatic artery with chemotherapeutic agents (Boursier et al. 2016).

**Table 3:** Comparison of mechanical properties of real human liver and silicone-based liver phantoms (Afiqah Bakri et al. 2019; Cafarelli et al. 2017)

Sample	Density (kg/m^3^)	Acoustic impedance (MRayl)	Attenuation (dB/cm/MHz)	Sound speed (m/s)
Human liver	1060	1.6	0.7	1540
Silicone and graphite mixture	1080	1.1	2.2	1080
Ecoflex0010	1063	1	1.5	1000

**Fig. 6:**
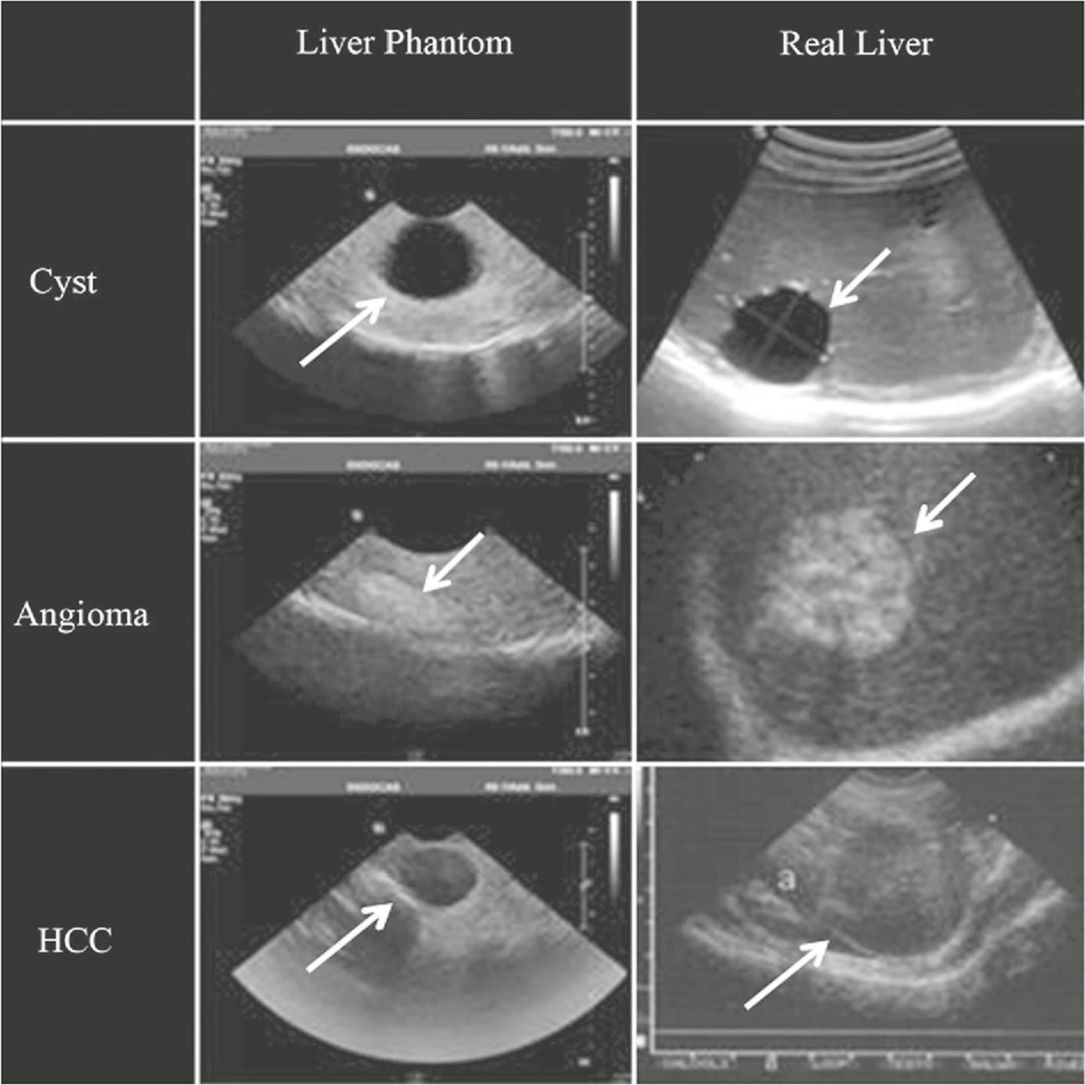
Lesions of anechoic, hyperechoic, and hypoechoic for phantom liver lesions (first column) and real liver organ (second column). Compared to the real mass, the anechoic mass ascribable to a cyst (first row), angioma (second row), and hypoechoic lesion simulating real hepatocellular carcinoma (HCC) (last row) (Pacioni et al. 2015), with permission of Springer Nature, copyright 2015

The differences in the mechanical properties of silicone-based phantoms mimicking the real liver organ are due to the amount of graphite used to improve the scattering agent (Makhamrah et al. 2019; Opik et al. 2012; Rafiq et al. 2018). Thus, adding slacker and thinner decreases the viscosity and enhances the homogeneity to overcome the mechanical and acoustic problems arising from the use of graphite (Zell et al. 2007). In addition, the thinner Vaseline oil allows better signal transmission (Zell et al. 2007). Figure 6 shows natural liver and liver phantom’s anechoic, hyperechoic, and hypoechoic lesions (Pacioni et al. 2015). The anechoic mass is ascribable to a cyst (Fig. 6, first row), a hyperechoic lesion simulates the angioma (Fig. 6, second row), and the hypoechoic lesion mimics the real HCC (Fig. 6, last row). Silicone-based mixtures develop the patient-specific liver phantom for ultrasound and biopsy hybrid simulators based on harmless, low-cost, and high-stability materials (Karimi and Shojaei 2018). In addition, such mixtures are the better 3D models to mimic the liver’s different lesions, vessels, and parenchyma, as demonstrated in Fig. 6 (Pacioni et al. 2015).

## Failure properties

**Fig. 7:**
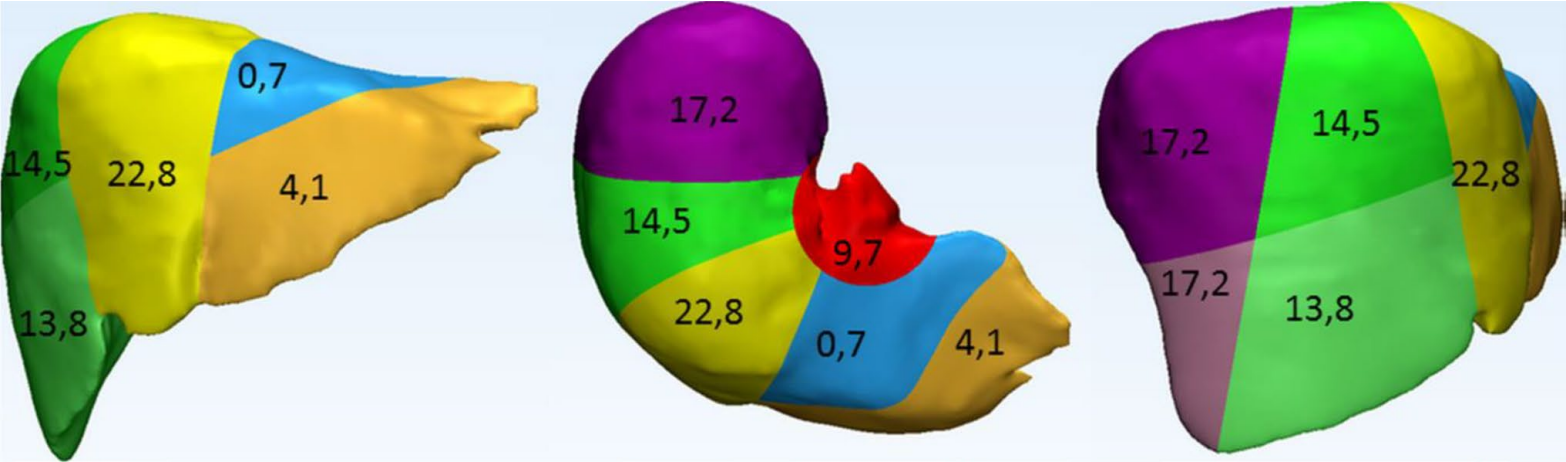
Injury percentage in the liver by segments due to vehicle accidents (Chenel 2018), with permission of Biomechanics Publications, copyright 2018

Many studies have investigated liver failure properties resulting from direct cellular damage, particularly in car accidents (Karimi and Shojaei 2018). The data show that vehicle crashes led to severe injuries across different liver segments requiring considerations in the protection assessments of vehicle passengers, as presented in Fig. 7 (Chenel 2018). This organ could undergo three types of injuries like, vascular, hematoma, and laceration failures, affecting the dense vascular network, capsule, and parenchyma, respectively (Chenel 2018; Mannelli et al. 2012). Severe injuries occur mainly due to the surface of the parenchyma during the accident (Mannelli et al. 2012).

**Fig. 8:**
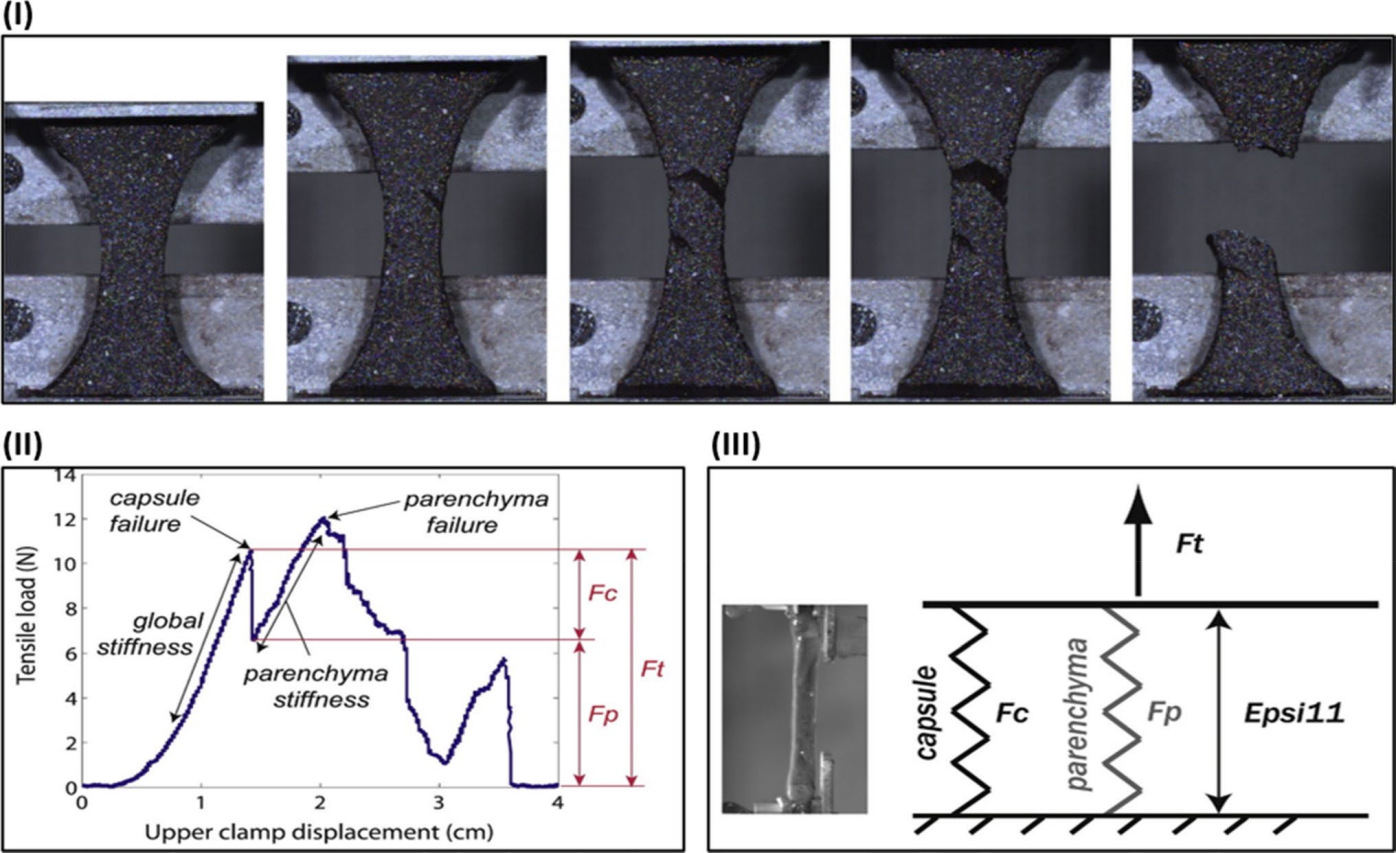
Mechanical failure analysis of the human liver. **I** Tensile test on the liver capsule and parenchyma sample leads to capsule failure and parenchyma. **II** The Resultant load–displacement curve and **III** the bi-material model consisting of two parallel springs (Brunon et al. 2010), with permission of Elsevier Ltd., copyright 2010

The mechanical properties used to analyze failure involve the ultimate tensile strain, true stress, and ultimate load per width (Brunon et al. 2010). During the tensile test, the mechanical failure mechanism emerged first on the capsule and then on the parenchyma, as displayed in Fig. 8I. The resultant load–displacement curve until the end of the capsule and parenchyma failures is shown in Fig. 8II. The capsule constitutive law is determined through the bi-material model of two parallel springs, as presented in Fig. 8III. The model splits the load measurements into capsule load (*F*_c_) and parenchyma load (*F*_P_) to investigate the sustaining load of each separately.

The liver capsule failure parameters are the ultimate load per unit width instead of maximum longitudinal stress due to its high thickness of 100 µm (Brunon et al. 2010). The normalized load is the slope of the load–displacement curve, indicating the load per unit width against the longitudinal strain (de Jong et al. 2019). The ultimate load is the proportion of sustained load via the liver capsule in the existing width about the localization area, known as true load per width unit (Mazza et al. 2008). Table 4 summarizes the mechanical failure properties of liver capsules for humans and porcine based on fresh and frozen samples (Brunon et al. 2010; Johnson et al. 2021; Opik et al. 2012). Then, we calculate the ultimate true stress and true modulus by dividing the ultimate load per width and normalized load, respectively, with average liver capsule thickness for porcine and humans (Basdogan 2012). The ultimate local strain is more precise than the global strain measurements due to the strain localization (Basdogan 2012). The normalized load in human fresh and frozen capsules is 2.02 ± 1.18 N/mm and 3.27 ± 2.70 N/mm, respectively. In contrast, the true modulus of the same samples is 16.9 ± 9.9 MPa and 27.5 ± 22.7 MPa, respectively, compared to frozen and fresh porcine capsule samples with 11.6 ± 19.2 MPa and 7.8 ± 10.5 MPa, respectively.

**Table 4:** Outcomes of the Mann–Whitney statistical test on failure mechanical properties of the liver capsule in human and porcine tissues (Brunon et al. 2010; Johnson et al. 2021; Opik et al. 2012)

	Fresh	Frozen
*Normalized load (N/mm)*		
Human	2.02 ± 1.18	3.27 ± 2.70
Porcine	1.44 ± 1.76	1.53 ± 2.08
*True modulus (MPa)*		
Human	16.9 ± 9.9	27.5 ± 22.7
Porcine	11.6 ± 19.2	7.8 ± 10.5
*Ultimate load per width (N/mm)*		
Human	0.22 ± 0.14	0.33 ± 0.32
Porcine	0.40 ± 0.48	0.24 ± 0.22
*Ultimate true stress (MPa)*		
Human	1.85 ± 1.18	2.77 ± 2.69
Porcine	2.03 ± 2.44	1.22 ± 1.12
*Ultimate strain (%)*		
Human	32.6 ± 13.8	43.9 ± 24.2
Porcine	43.3 ± 25.4	62.9 ± 35.4

The principal statistical analysis in the literature of human liver capsules’ failure behavior and properties shows a strong dependence on the fresh or frozen hepatic tissue state. For instance, the ultimate strain of the porcine capsule is strongly altered by the freezing as it damages its hepatic tissues more than the human capsule, as displayed in Table 4. The conclusions in the literature on the influence of freezing on mechanical properties vary for soft biological tissues such as a capsule, parenchyma, ligaments, and muscle (Afiqah Bakri et al. 2019). The cause is probably the widely different constitutions and mechanical loadings (Afiqah Bakri et al. 2019). Considering the fresh or frozen liver status is significant in developing liver phantoms investigating the severely rejected transplanted livers from the non-rejected ones, which might be frozen before being transported.

## Mechanical modeling and simulation of liver tissues to develop mimicking phantoms

The liver constitutive formulation anticipates merging the polynomial and logarithmic strain energy in modeling united elongation and compression experiments on the hepatic tissue (Rethy et al. 2018). The heterogeneous structure of liver tissues has caused most of its published mechanical models in the literature to be nonlinear tissue constitutive equations (Capilnasiu et al. 2020; Chatelin et al. 2011). Instead, the models attempt to compute the bulk tissue mechanical properties and time constants (Yarpuzlu et al. 2014). The published data highlight the differences in tissue conditions like aging or pathophysiological state (Yarpuzlu et al. 2014). Therefore, it is only meaningful to compare parameters from the same constitutive model.

**Fig. 9:**
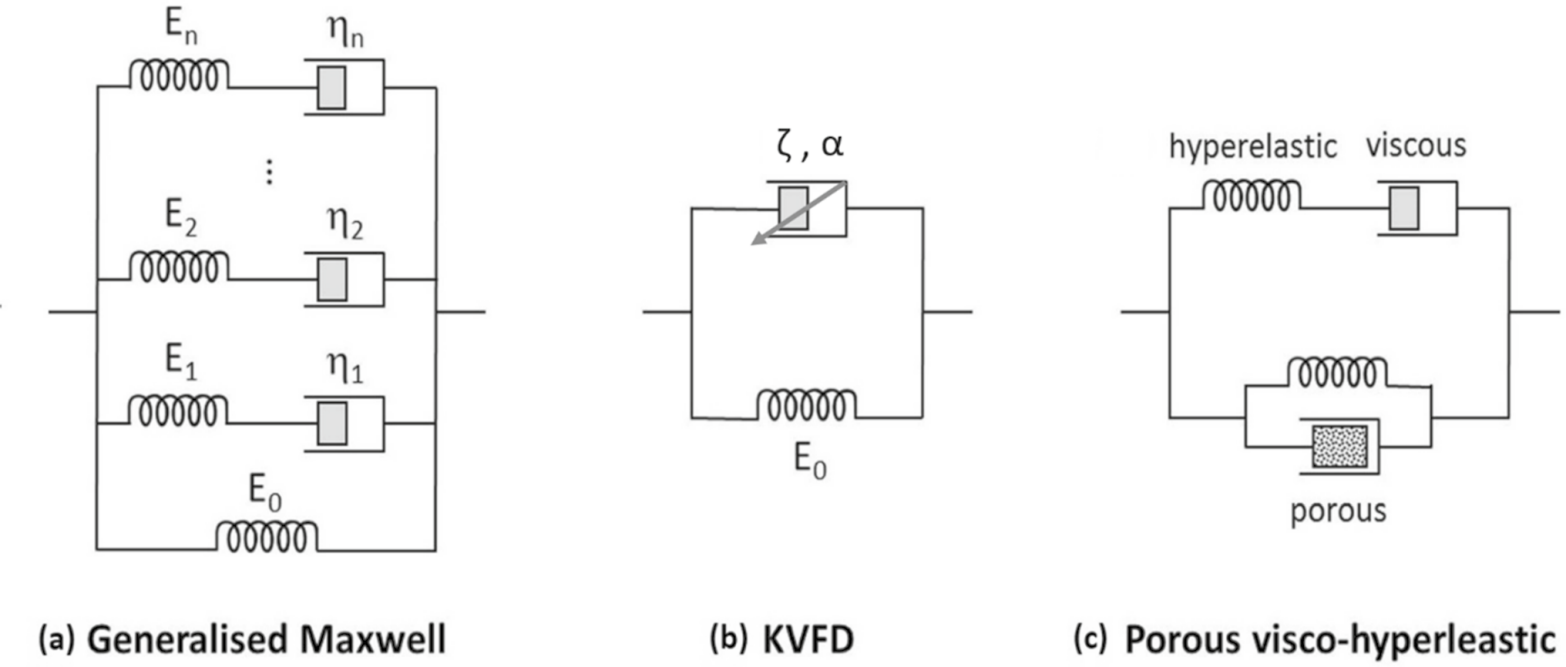
Basic models describing the mechanical properties of the liver: **a** generalized Maxwell, **b** Kelvin–Voigt fractional derivative (KVFD), and **c** porous visco-hyperelastic (Mattei and Ahluwalia 2016), with permission of Elsevier Ltd., copyright 2016

The choice of tissue model representing the liver’s mechanical behavior still varies among scientists (Lin et al. 2017). However, due to the prominent viscoelastic behavior, the three standard models are the generalized Maxwell (GM), the Kelvin–Voigt fractional derivative (KVFD), and the porous visco-hyperelastic models, as illustrated in Fig. 9 (Mattei and Ahluwalia 2016). The following sections will detail each model separately to better visualize their principle in fitting the liver mechanical data in comparison with other common models, namely standard linear solid (SLS).

## Generalized Maxwell model

By using viscous (dashpots, *η*_i_) and elastic (springs, *E*_i_) parts for the most general form utilized in modeling the linear viscoelastic behavior of soft tissues like the liver based on Maxwell’s arms (*n*), a spring and dashpot in series, in parallel connections with a spring are demonstrated in Fig. 9a (Mattei and Ahluwalia 2016). The dashpot is the energy dissipative element, and the spring is the energy storage element. The springs represent the flexible component of the model’s response and obey Hook’s law and generate stress (*σ*_spring_) proportional to strain (*ε*) using Eq. (1) (Evans and Gentleman 2014), while dashpots represent the viscous component of the viscoelastic hepatic tissues, with stress (*σ*_dashpot_) being proportional to the strain rate ($\dot{\varepsilon }$), as shown in Eq. (2) (Zhu et al. 2016).

1 $${\sigma }_{\mathrm{spring}}= E\varepsilon $$

2 $${\sigma }_{\mathrm{dashpot}}= \eta $$

*E* and* η* are Young’s modulus and the viscosity of the liver, respectively (Zhu et al. 2016). The total strain of the Maxwell model is the sum of the strains in the dashpot and spring components. Equation (3) shows the constitutive relation of the Maxwell model expressed as a linear first-order differential equation in a function of the two structural constituent parameters (*E* and *η*) (Pasyar et al. 2020):

3 $$\sigma (t)+ \frac{\eta }{E}\frac{\partial \sigma (t)}{\partial t}= \eta \frac{\partial \varepsilon (t)}{\partial t}$$

**Fig. 10:**
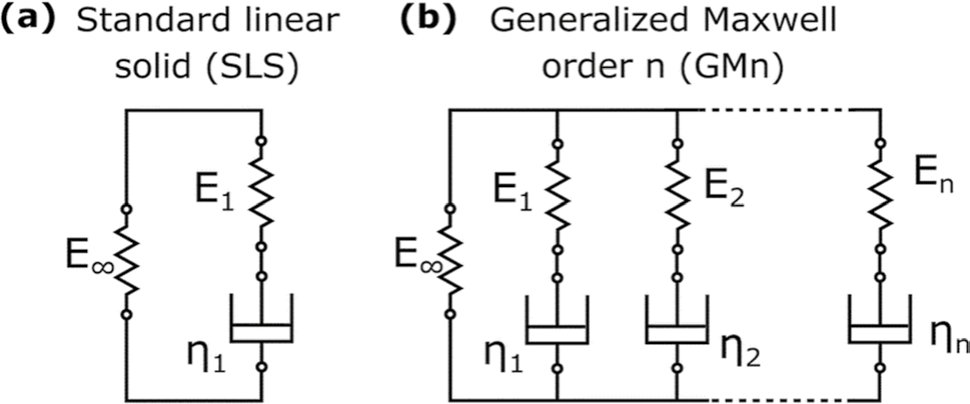
Comparison between the **a** standard linear solid (SLS) with three parameters and **b** generalized Maxwell with 2n + 1 parameters and order n for modeling the mechanical properties of the liver (Ganser et al. 2017), with permission of the Royal Society of Chemistry, copyright 2018

A simpler linear form, known as the standard linear solid (SLS) or Zener model, simplifies the generalized Maxwell model but with only one spring–dashpot branch, as illustrated in Fig. 10 (Ganser et al. 2017). The order (*n*) of the generalized Maxwell model indicates the order of the resulting differential equation for strain and stress. Consequently, the SLS model is a generalized Maxwell model of order 1 and differential equation (Klatt et al. 2010):

4 $$\sigma \left(t\right)+ \frac{{\eta }_{1}}{{E}_{1}}\frac{\partial \sigma \left(t\right)}{\partial t}= \eta \frac{\partial \varepsilon \left(t\right)}{\partial t}+{E}_{\infty }\varepsilon \left(t\right)+\frac{{\eta }_{1}{E}_{\infty }}{{E}_{1}}\frac{\partial \varepsilon \left(t\right)}{\partial t} $$

The SLS model is used to predict the strain curve and the behavior for instantaneous loads and, long time, the model deficiencies in accurately modeling material systems numerically (Zhu et al. 2014).

## Kelvin–Voigt fractional derivative (KVFD) model

**Fig. 11:**
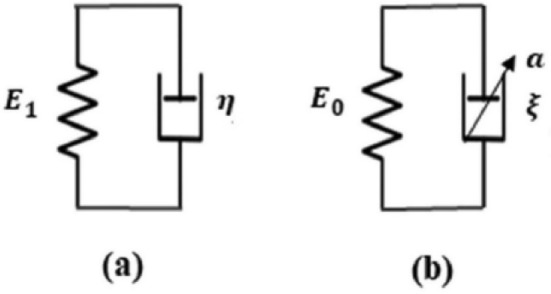
Comparison between the **a** Kelvin–Voigt (KV) model with two-parameter and **b** Kelvin–Voigt fractional derivative (KVFD) model with three parameters for modeling the mechanical properties of the liver (Poul et al. 2022), with permission of Elsevier Ltd., copyright 2022

It models the quasi-linear or nonlinear behavior of biological tissues, which have a nonlinear stress–strain relationship, as displayed in Fig. 9b (Mattei and Ahluwalia 2016). Such nonlinearity could inhibit assessments of various studies on living tissue mechanics (Kassner et al. 2009). Kelvin–Voigt (KV) model is a two-parameter model involving a spring with Young’s modulus (*E*_1_) and a dashpot element with viscosity (*η*) connected in parallel, as shown in Fig. 11a (Poul et al. 2022). The generalization of the KV model is the KVFD model, with the stress (*σ*) in the dashpot being equivalent to the fractional derivative of order α for the strain (*ε*) (Mattei and Ahluwalia 2016). A fractional derivative approximates the derivative of a function to a real number order α,

5 $$\sigma \left(t\right)=\upeta \frac{{\partial }^{\alpha }\varepsilon (t)}{\partial {t}^{\alpha }}$$

It is represented by a spring-pot (fractional dashpot), as illustrated in Fig. 11b (Poul et al. 2022). When *α* = 1, the spring-pot behaves as a dashpot element; for *α* = 0, it acts as a spring (Hafsah et al. 2014).

The KVFD model is:

6 $$\sigma \left(t\right)= {E}_{0}\varepsilon \left(t\right)+\upeta \frac{{\partial }^{\alpha }\varepsilon (t)}{\partial {t}^{\alpha }}$$

where *α* ranges between 0 and 1; the *E*_0_ and* η* are the spring elastic constant and viscosity coefficient of the dashpot, respectively (Mattei and Ahluwalia 2016). The relaxation time constant (*τ*) is used instead of* η* by letting* η*= E_0_τ^α^, resulting in Eq. (7) (Mattei and Ahluwalia 2016). The form of this equation has three constants characterizing the materials’ mechanical behavior (i.e., *E*_0_, *τ*, and *α*) (Marchesseau et al. 2017).

7 $$\sigma \left(t\right)= {E}_{0}\left(\varepsilon \left(t\right)+ {\tau }^{\alpha }\frac{{\partial }^{\alpha }\varepsilon (t)}{\partial {t}^{\alpha }}\right)$$

The KVFD has the following significant differences (Poul et al. 2022; Zhu et al. 2016):

i. KVFD has a continuous and gradual response in creep compliance, whereas the SLS has a discontinuous and instantaneous response at time zero.
ii. Stress relaxation varies at *t*^−α^ for the KVFD model but decays exponentially in the SLS model.
iii. In the KVFD model, the frequency response of the complex Young’s modulus is dependent on ωα instead of only ω (where ω is the angular frequency).

The frequency response in the KVFD model undergoes a monotonically increase dissimilar to that in the SLS model, attaining a plateau (Glińska-Suchocka et al. 2017; Pi et al. 2021). In addition, the measured velocity during the propagation of shear waves across the liver at distinct values ranging from 40 Hz to 14 MHz indicates the monotonical increase of shear velocity with frequency (Glińska-Suchocka et al. 2017; Pi et al. 2021). Concluding from the dynamic testing on tissues of the canine liver, the KVFD model exhibited a good fit with the experimental data compared to other models, such as the KV model (Yeh et al. 2015).

The KVFD model has been used frequently for assessing liver viscoelasticity, as reported in most studies (Evans et al. 2013; Untaroiu et al. 2015). A recent study has conducted rheological experiments on rat livers to quantify their mechanical behavior at various steatosis stages (Yilmaz 2020). Zener, Maxwell, and Kelvin–Voigt models have been used to analyze mechanical properties. The model has to satisfy both the ex vivo dynamic mechanical analysis (DMA) experiment at low frequency (1–41 Hz) and the in vivo shear wave elastography (SWE) experiment at high frequency (160–380 Hz) (Pellot-Barakat et al. 2016). Table 5 summarizes the fitness profile for each of the three models to the shear wave velocity (Lin et al. 2017; Pi et al. 2021). The tabulated results reveal that the best model characterizing the rats’ mechanical properties at every steatosis stage is the Voigt model with a determination coefficient (*R*^2^) near 1.

**Table 5:** Fitting effect of the three common models in dynamic mechanical analysis (DMA) and shear wave elastography (SWE) experiments on normal and steatosis liver tissues, with n being the number of rat samples. (Lin et al. 2017; Pi et al. 2021)

Model	The determination coefficient (*R*^2^)			
	SWE		DMA	
	Normal (*n* = 3)	Steatosis (*n* = 3)	Normal (*n* = 6)	Steatosis (*n* = 6)
Generalized Maxwell	0.35	0.35	0.52	0.53
KVFD	0.80	0.84	0.89	0.88
Zener	0.54	0.44	0.94	0.92

## Porous visco-hyperelastic model

The liver tissues are modeled frequently as a fluid-filled porous matrix. This liver model includes viscosity, porosity, and hyperelasticity, modeled by the Prony series (Payan and Ohayon 2017). At the same time, the linear Darcy law signifies the mechanical impact of liver perfusion based on the porosity model working parallel to visco-hyperelastic components, as demonstrated in Fig. 9c (Mattei and Ahluwalia 2016). The poro-visco-elasticity (PVE) model extends the biphasic theory and describes tissues into two phases of immiscible mixtures containing incompressible phases of the inviscid fluid and solid elastic phases (Chmarra et al. 2013). The solid phase has intrinsic viscoelasticity, considered in the flow-independent viscoelastic behavior. In contrast, its solid phase has been modeled as a viscoelastic material using a Prony series with *n* = 3 Maxwell arms (spring–dashpot) parallel to a single spring (Mattei and Ahluwalia 2016). The testing of ex vivo porcine liver specimens at different ramp strain rates ranging from 0.001 s^−1^ to 0.1 s^−1^ has shown that the use of the viscoelastic (VE) model resulted in an underestimating of the peak force values in contrast to the PVE model, which is due to the absence of a fluid phase (Chen and Shih 2013).

**Fig. 12:**
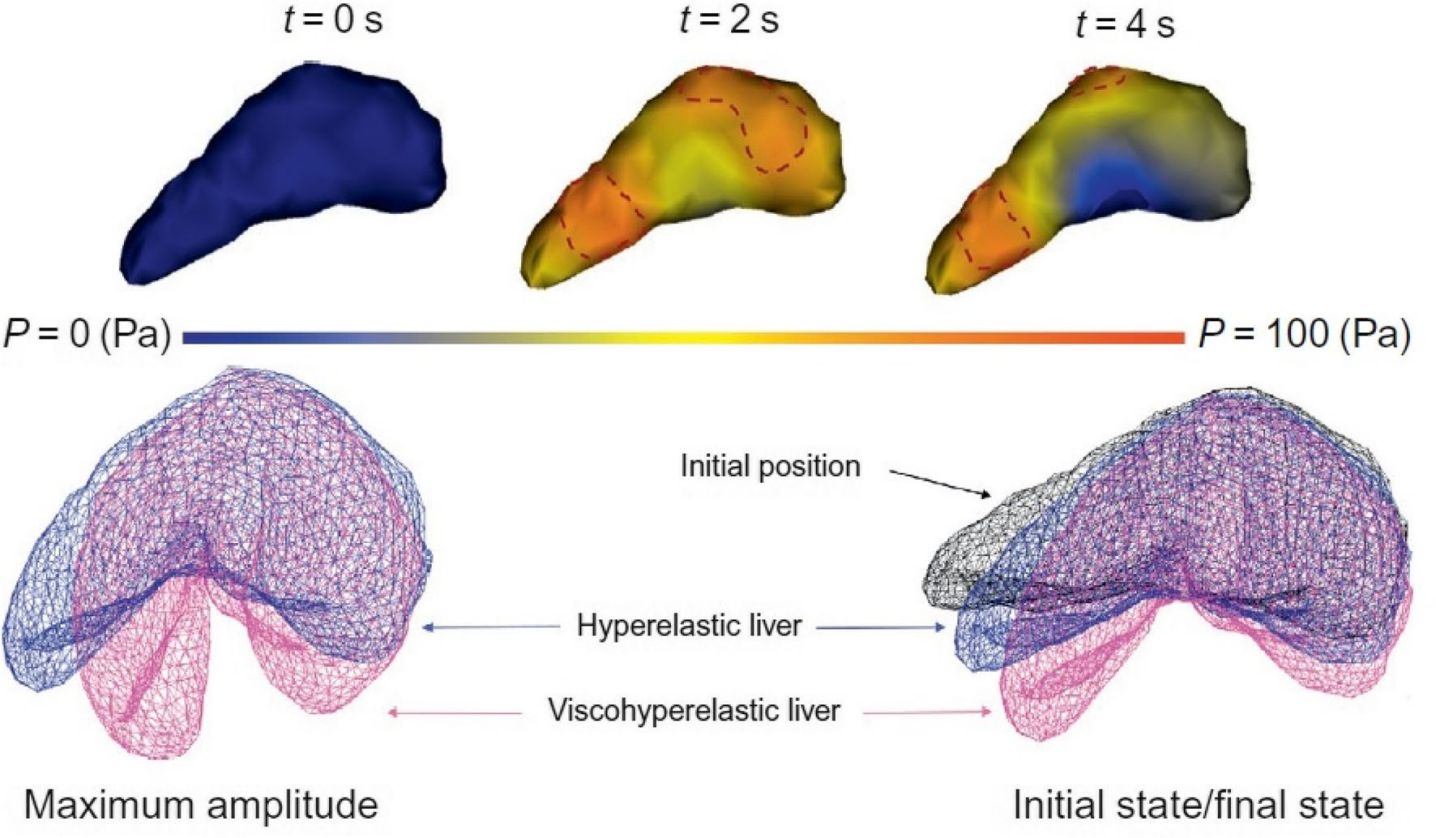
(Top) Pressure field of the liver porous component at the action of gravity (dotted lines highlight the highest-pressure areas). (Bottom) The Counting of viscosity to hyperelasticity with a comparison of the initial/final states and maximum amplitude (Marchesseau et al. 2017), with permission of Elsevier Ltd., copyright 2017

Investigating the influence of the viscous component by adding viscosity to hyperelasticity will increase the liver's amplitude as the material's stiffness increases (Rus et al. 2020). The final state shows the difference in the final state with respect to the visco-hyperelastic compared to hyperelastic models, as demonstrated in Fig. 12 (bottom) (Marchesseau et al. 2017). Under the action of gravity, the liver deforms to overpass the linearity bound of the material as a large amount of compression and extension can occur due to the liver’s porous component controlling the viscosity quantity (Idilman et al. 2020). The implicit integration scheme enables more significant time steps like 0.3s, making the real-time interaction probable (Marchesseau et al. 2017). Figure 12 (top) is a color map of the fluid pressure field simulated throughout the deformation, which ranges from the initial pressure (dark blue) to the highest pressure (red) (Marchesseau et al. 2017). A comprehensive model combining porosity and visco-hyperelasticity prevents the liver from undergoing unrealistic deformations (Nava et al. 2008). Consequently, the deformation is not homogenous any longer and varies with time.

## Computational simulation

Finite element (FE) is a method for numerically solving differential equations on a structured mesh representing physical geometries (Brock et al. 2002). The FE modeling has been used to aid surgical decisions by providing simulated outcomes in real time through augmented reality (Michaël Kugler et al. 2018a, b). The real-time organ simulation through FE modeling requires precision and time efficiency to enhance patient-specific modeling, which could be utilized in surgical environments (Chanthasopeephan et al. 2007).

**Fig. 13:**
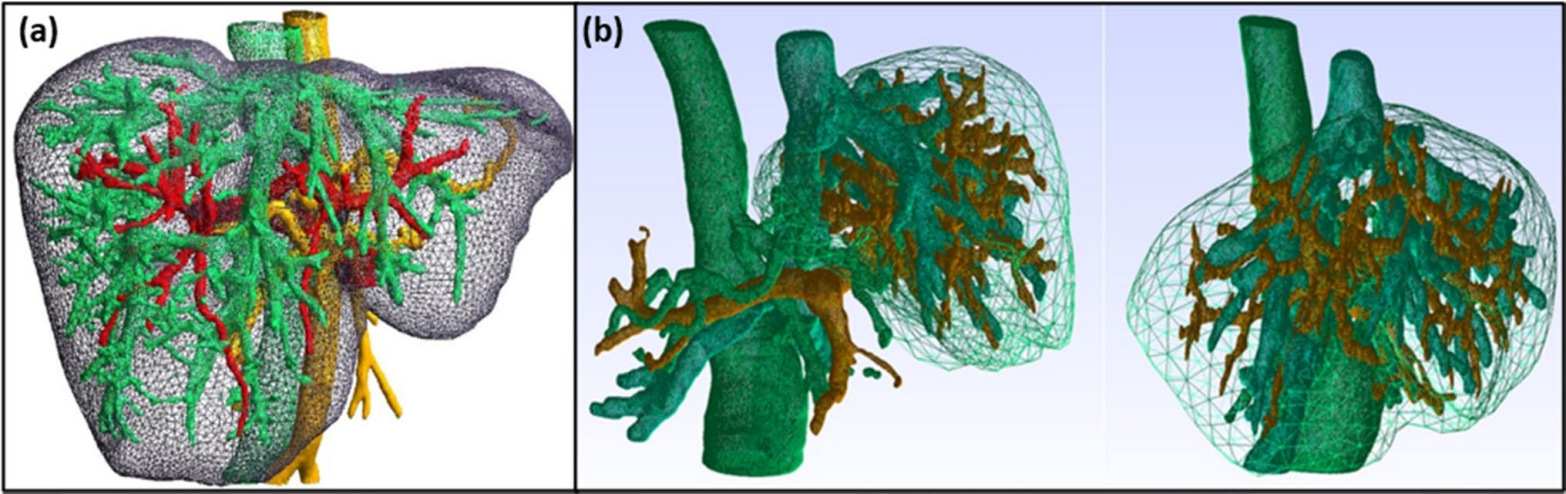
**a** The FE modeling of liver geometry and its vascularization with each cube representing a heterogeneous segment (Kugler 2018), with permission of HAL archives-ouvertes.fr, copyright 2018. **b** Example of liver and vascularization surface meshes obtained through segmentation of CT scan images from IRCAD with light mesh corresponding to the liver and other brown, blue-green, and green dense meshes corresponding to the vascularization (Michael Kugler et al. 2018a, b), with permission of Elsevier Ltd., copyright 2018.

Additionally, simulating hyperelastic models is significantly cheaper than most viscoelastic models (Hashemi et al. 2020). The constitutive models of soft tissues rely mainly on the homogenized, anisotropic, and hyperelastic model mimicking the response of the actual hepatic tissues (Hashemi et al. 2020). The heterogeneity of the liver tissue impacts the real-time biomechanical response in augmented reality surgery (Kumar 2015). Figure 13a shows the mesh geometry of the liver and its vascularization based on its dissimilar segmentations, which demand merging for a complete 3D anisotropic structure (Kugler 2018). Michael Kugler et al. applied more complex liver meshes and counted their vascularization, as shown in Fig. 13b (Michael Kugler et al. 2018a, b). The generated geometries and overlapping meshes are from medical images such as CT scan and MRI (Chi et al. 2011). The vascularization is integrated into a single mesh to perform the mechanical simulation (Lauzeral et al. 2019).

Chi et al. considered 20 patient livers and vascularization extracted from the IRCAD open database in FE modeling (Chi et al. 2011). The complexity of the vascularization geometries shows significant variations in the mesh precision compared to the patient livers and in the vascularization density varying from 0.8% to 3.3%, with an average of 1.4% of the total liver volume (Chi et al. 2011). Thus, there is a need for an intelligent homogenized model considering the anisotropic and nonlinear behavior of the liver to integrate it within real-time computations (Untaroiu and Lu 2013). The combination of medical imaging techniques and precise quantification of the liver's mechanical properties enables accurate patient liver simulations, consideration of the inner vascular distributions, and probable tumors utilizing noninvasive in vivo characterization (Cheng and Hannaford 2015).

## Fabrication materials to develop liver-tissue-mimicking phantoms

The fabrication material of the liver phantom seeks to mimic the structure and morphology of the actual liver in the human body. Different materials are developed to obtain an ideal liver phantom with long-term stability for liver procedures (Ahmad et al. 2020a, b). The fabrication materials of the liver's phantom should be safe to prepare and handle, stable under different environmental conditions, facile and reproducible in preparation, easy to store and transport, and demand low-cost ingredients (Mattei et al. 2022). Table 6 displays the mechanical properties of the commonly utilized fabrication materials of liver phantoms, including styrene-ethylene-butylene-styrene (SEBS) copolymer, gelatin, polyvinyl alcohol (PVA), silicone, polyvinyl chloride (PVC), and agar. The mechanical properties per material are compared with the hepatic tissues and scored on their closeness. The durability property includes shelf life, resistance to deformation/cracking, and storage requirements.

**Table 6:** Mechanical properties of common fabrication materials for phantoms mimicking the human liver

Fabrication material	Speed of sound (m/s)	Attenuation (dB/cm/MHz)	Young’s modulus (kPa)	Durability	Refs.
Styrene-ethylene-butylene-styrene (SEBS) copolymer	++(1423–1502)	+(0.25–0.42)	+(26–70)	+++	Ahmad et al. (2021), Cabrelli et al. (2017)
Gelatin	+++(1510–1590)	+++(0.12–1.53)	+++(35–58)	+	Anugrah et al. (2020), Kandala et al. (2021)
Polyvinyl alcohol (PVA)	+++(1520–1610)	+(0.07–0.35)	++(60–125)	+	Cournane et al. (2010), In et al. (2012)
Silicone	+(1000–1150)	+(1.25–2.63)	++(25–82)	+++	Chen and Shih (2013), Lamouche et al. (2012)
Polyvinyl chloride (PVC)	++(1400–1420)	++(0.44–0.65)	+++(24–123)	+++	Chatelin et al. (2020b), Rethy et al. (2018)
Agar	+++(1540–1600)	+++(0.04–1.42)	+(105–115)	+	Ahmad et al. (2022), In et al. (2014)

+ = worst;++ = suitable;+++ = best

The most frequently reported property is the speed of sound owing to its significance for ultrasound phantoms in calibrating clinical transducers to be close to the typical value of hepatic tissues (1540 m/s). Varying the concentration of backscatter agents can increase the attenuation coefficient with negligible effect on the speed of sound (Ahmad et al. 2020a, b). Considering both attenuation and speed of sound, gelatin and agar closely mimic biological tissue and produce realistic ultrasound images for building anatomical structures of varying compositions. The ultrasound scans of PVA are reported with pixel intensity close to human tissues (Cournane et al. 2010). The fabrication method of PVA is long and complex (Cournane et al. 2010). Still, varying material composition and the number of freeze–thaw cycles (FTCs) might tune its acoustic and mechanical properties to mimic hepatic tissues (de Jong et al. 2019). On the other hand, silicone is anechoic on ultrasound due to its high attenuation and low speed of sound compared to biological tissues (Ansar et al. 2019). PVC also has a speed of sound below that of hepatic tissues, which can be notably improved with appropriate plasticizer selection and concentration (Chatelin et al. 2020a).

Considering the tactile feedback resulting from a suitable mechanical response of the phantom, fabrication materials such as PVC, PVA, and gelatin revealed tunability mimicking the soft tissues' elasticity (Ahmad et al. 2020a, b). Advanced shear wave speed technologies accurately represent tissue elasticity compared to the longitudinal measurement of Young's modulus, which is limited by tissue anisotropy (Nikolaev and Cotin 2020). An additional advantage of silicone, SEBS, and PVC materials is being insoluble in water (Mattei et al. 2022). As a result, phantoms fabricated from these materials show more stability and durability throughout aging (Mattei et al. 2022). In contrast to phantoms fabricated from hydrated materials, including gelatin and agar, which dehydrate or foster bacterial growth (Łabowska et al. 2021). The less traditional tissue-mimicking materials such as zerdine, urethane, polyacrylamide (PAA), household items (e.g., hair gel, condensed milk, wire-pulling lubricant), and foodstuffs (e.g., chicken breast, mixed-meat rolls, and tofu) are reported as low-cost vascular access phantoms but with limited re-usability, short shelf-life, and absence of studies onto their mechanical properties (McGarry et al. 2020).

Eventually, there might be a need for a compromise between the phantom’s requirements in terms of fabrication, storage, mechanical feedback, and acoustic properties. In phantoms’ fabrication, the selection of the tissue-mimicking material should be based on carefully considering its fabrication purpose. For instance, PVC exhibits good shelf-life and mechanical properties suitable for prolonged use (Chatelin et al. 2020b). Alternatively, gelatin and agar have tissue-like ultrasound compatibility for quantitative measurement of attenuation and speed of sound (Chen et al. 2022). If storage requirements and tedious fabrication methods can be overcome, PVA is the best option due to its excellent tunability for all demanded properties. Future phantoms might mimic the complex structure of human hepatic tissues by combining existing tissue-mimicking materials or fabricating multiple tissue layers to investigate spatial control of acoustic and mechanical properties (Stengl et al. 2022). Additive manufacturing can assist in anatomical landmarks and raise the fidelity of phantoms mimicking human hepatic tissues (Stengl et al. 2022). For example, 3D-printed phantoms based on silicone with additive materials, such as water glucose solution and tertbutyl, have been utilized to mimic specific tissues like fatty liver tissues (Morr et al. 2021b).

## Challenges and outlook

Precisely characterizing the liver’s mechanical behavior is pertinent in in vitro applications, diagnostic purposes, and tissue engineering (Chatelin et al. 2011). The modeling and quantifying of materials’ mechanical properties are necessary to understand, monitor, and predict their responses and performance under certain loading conditions. The mechanical characterization through constitutive modeling demands identifying and controlling environmental and geometric testing boundary conditions. For decades, characterizing structural materials have been done through different testing approaches. However, reliable data for degradable and hydrated soft materials are still deficient, particularly the non-load-bearing biological tissues, including the kidney, brain, and liver (Afiqah Bakri et al. 2019). The primary reason is their softness, shape, and labile nature (Afiqah Bakri et al. 2019). In addition, such materials are biphasic, involving a solid network being completely swollen and bounded with liquid media (Huerta-López and Alegre-Cebollada 2021).

The mechanical behavior of biological tissues is characterized in vivo or ex vivo through numerous models and methods depending on direct tissue specimen measurements or image techniques (Mattei and Ahluwalia 2016). The testing of tissue in vivo preserves its status, but it has several constraints, including accessibility, subjecting humans to potential risks, and ethical issues regarding using animals (Alshipli et al. 2018; Makhamrah et al. 2019). Nevertheless, systematic studies have described and characterized the tissue mechanical behavior in vivo with datasets frequently restricted to minor deformations (Mazza et al. 2007). Furthermore, the in vivo data interpretation is also tricky due to the incapability to regulate the internal condition of the organ and challenges in finding a suitable arrangement for positioning the tested specimen and instrument (Crescenzi et al. 2019; Glińska-Suchocka et al. 2017).

Alternatively, the ex vivo trials are desirable in advancing novel testing tools, tissue models, and procedures to permit more direct and accessible testing trials fitting regulation of boundary conditions and have less ethically problematic compared to in vivo measurements (Gerhard et al. 2012; Johnson et al. 2021). Though the numerous published methods and studies in the literature, there still needs to be unconditional mechanical properties of the liver. The published results depend strongly on variations in testing protocols and techniques, as well as the differences in sample source, type, and status, which rely on the different objectives and needs of the researchers. Furthermore, implementing other tissue models as purely elastic models instead of poro-viscoelastic or viscoelastic models could influence the assessed hepatic tissue properties.

Moreover, stress relaxation and creep tests are the widespread measuring techniques for viscoelastic materials’ time-dependent behavior of liver tissues (Cai et al. 2017). Such tests have been utilized in combination or separately, enabling complete and precise data on the time-dependent behavior of viscoelastic samples (Bartolini et al. 2018). However, these tests demand the formation of initial contact between the testing apparatus and the sample allowing the initiation of the measurements (Bartolini et al. 2018). Consequently, it could lead to noteworthy pre-loading on the highly soft hepatic specimen and then changing its status. For instance, in crash simulation, the mechanical outcomes from impact tests have high strain rates (Untaroiu et al. 2015). Therefore, establishing standard protocols and tailored guidelines for data analysis, mechanical testing, and sample preparation for each application is necessary to facilitate comparative studies (Labonte et al. 2017; Mattei and Ahluwalia 2016).

Furthermore, there is a need for biomarker assessments in the safe and non-invasive characterization to replace the histological analysis relying on liver biopsy in quantifying and staging liver diseases and their progression from ongoing inflammation to consequent fibrosis. The liver disease stages correlate to their mechanical properties, which are sensitive to tissue alterations. Then, their assessment could be utilized as an alternative biomarker to the information from the liver biopsy test (Wells and Liang 2011). For instance, the fibrosis stages are correlated to liver stiffness, while the inflammation level is related to liver viscosity (Leitão et al. 2017). In addition, the diagnosis performance of liver steatosis staging improves by correlating the staging steatosis to the liver sound speed and attenuation (Casciaro et al. 2009). Thus, the stages of liver inflammation, liver steatosis, and liver fibrosis are of significant importance from a clinical perspective to monitor the NASH, antiviral, and antifibrotic treatments (Gidener et al. 2021), in addition, to follow up on the evolution of chronic liver diseases and assess their prognosis (Yin et al. 2011).

Additionally, considering liver tissue’s mechanical properties is an ultimate goal in manufacturing liver phantoms, with mechanics recapitulating the qualities of living tissue (Afiqah Bakri et al. 2019). Initially, mimicking the stiffness or/and softness of native liver structures requires considering their static mechanical properties (Rethy et al. 2018). However, the biological tissues have a dynamic nature leading to a necessity to integrate the mechanics’ changes in time/rate dependent and heterogeneity (Guimarães et al. 2020). Considering the time-varying mechanics by adopting materials from stress-relaxation or stress-stiffening responses mimicking the real hepatic tissue response is possible (Rafiq et al. 2018). Thus, the material will respond to the increased stress or continued duration through relaxation or stiffness, respectively (Makhamrah et al. 2019). The lacking material for such dynamicity could be overcome through the direct application of mechanical deformation on constructs to duplicate these properties. The heterogeneity-changing mechanics occurs in the tissue interfaces as in areas with cartilage turning progressively into bone (Khogalia et al. 2020). Mimicking these gradually varying structures is via gradients of mechanical properties, composition, and design (Khogalia et al. 2020).

## Conclusions

The liver phantoms are artificial structures designed to mimic the natural hepatic tissue properties, including their mechanical properties. Considering patient safety, these phantoms are utilized to fill the gap between theory and clinical practice for medical training and simulation. The fabrication materials of the liver's phantom should be safe to prepare and handle, stable under different environmental conditions, facile and reproducible in preparation, easy to store and transport, and demand low-cost ingredients. The constitutive models of mechanical properties for the viscoelastic hepatic tissues are generalized Maxwell (GM), standard linear solid (SLS), Kelvin–Voigt (KV), Kelvin–Voigt fractional derivative (KVFD), and porous visco-hyperelastic models. The finite element (FE) method is used in computational simulation to aid surgical decisions by providing simulated outcomes in real time through augmented reality. The mechanical properties, including elasticity, viscoelasticity, acoustic impedance, and attenuation, are further considered critical biomarkers for diagnosing different histopathologic scores of hepatic fibrosis, inflammation, and fat content in a patient without invasive biopsies. Future phantoms might mimic the complex structure of human hepatic tissues by combining existing tissue-mimicking materials or fabricating multiple tissue layers to investigate spatial control of acoustic and mechanical properties. Additive manufacturing can assist in anatomical landmarks and raise the fidelity of phantoms mimicking human hepatic tissues.

## Abbreviations

TMMs: Tissue-mimicking materials
HCC: Hepatocellular carcinoma
MRI: Magnetic resonance imaging
4D CT: Four-dimensional computed tomography
PVC: Polyvinyl chloride
PVA: Polyvinyl alcohol
PAA: Polyacrylamide
SEBS: Styrene-ethylene-butylene-styrene copolymer
E: Young’s modulus
E′: Viscoelastic storage modulus
E″: Viscoelastic loss modulus
ASTM: American Society for Testing and Materials
G′: Shear storage modulus
G′′: Shear loss modulus
USWE: Ultrasonic shear wave elastography
MRE: Magnetic resonance elastography
AMUSE: Attenuation measuring ultrasound shear wave elastography
KVFD: Kelvin–Voigt fractional derivative model
SLS: Standard linear solid model
KV: Kelvin–Voigt model
DMA: Dynamic mechanical analysis
SWE: Shear wave elastography
PVE: Poro-visco-elasticity theory
FE: Finite element method
SSI: Supersonic shear imaging
SWS: Shear wave speed
NAFLD: Non-alcoholic fatty liver disease
ELF: Enhanced liver fibrosis
ECM: Extracellular matrix
